# STEPS: A Solution for Ensuring Standards of TB Care for Patients Reaching Private Hospitals in India

**DOI:** 10.9745/GHSP-D-20-00449

**Published:** 2021-06-30

**Authors:** Shibu Balakrishnan, Rakesh PS, Sunilkumar M, Bhavan Sankar, Rakesh Ramachandran, Ameer KA, Ramani Gopi, Prem Nair

**Affiliations:** a World Health Organization, National TB Elimination Program Technical Support Network, Cochin, Kerala, India.; b State TB Cell, Thiruvananthapuram, Kerala, India.; c IMA END TB Project, Kerala, India.; d Project JEET, State TB Cell, Kerala, India.; e Coalition of Professional Medical Associations for TB Elimination, Kerala, India.; f Kerala Institute of Medical Sciences, Thiruvananthapuram, Kerala, India.; g Infection Control & STEPS, Renai Medicity, Cochin, Kerala, India.; h Amrita Institute of Medical Sciences, Cochin, Kerala, India.

## Abstract

**Background:**

In India, the private sector diagnoses and treats more patients with TB than the public sector. Gaps in the TB care cascade were observed more among the patients diagnosed in the private sector.

**Concept:**

The System for TB Elimination in Private Sector (STEPS) model evolved as a solution to address gaps in the quality of care for patients in the private sector by ensuring standards of TB care. STEPS has 3 components: a consortium of private hospitals, a coalition of all professional medical associations, and a STEPS center in each private hospital. STEPS centers act as a single window for notification, linkage for social welfare measures, contact investigation, chemoprophylaxis, direct benefit transfers, and treatment adherence support.

**Intervention:**

STEPS was piloted in 14 districts in the state of Kerala. All 14 districts formed consortiums of private hospital management for policy support and a coalition of professional medical associations for advocacy with doctors. STEPS centers were established in 318 private hospitals.

**Results:**

Notification to National TB Elimination Program from the private sector improved by 26% when compared to the previous year. Among the patients notified from the private sector, microbiologically confirmed cases increased by 81%, rifampicin resistance testing at baseline increased by 56%, and the percentage of those informed of their HIV status increased by 95%. The percentage of patients notified from the private sector with their treatment outcome reported improved from 39% (2018) to 99% (2019).

**Conclusion:**

The STEPS model demonstrated that a low-cost locally customized private sector engagement model is feasible and is beneficial to society. STEPS could be one of the major solutions for supporting patients reaching the private sector.

## Abstract (precis)

A low-cost model for engaging the private sector to address gaps in TB care and ensuring that patients in the private sector receive the standards of care in India was feasible. The pilot project showed improvements in standards of care, which benefits the patient, government, private hospitals, and society.

## Teaser

**Key Findings:**

- The STEPS (System for TB Elimination in Private Sector) model demonstrated that a “patient-centric” strategy where private hospitals follow up their patients with TB for clinical actions and public health actions is feasible.
- STEPS successfully addressed the gaps in the quality of care for patients seeking care in the private sector and ensured that services are aligned with the standards of TB care.
- STEPS model helped the state government in establishing a good surveillance system for TB within private hospitals.

**Key Implications:**

- Policy makers may consider conceiving and implementing locally customized models based on the principles of STEPS for ensuring standards of care to TB patients seeking care from the private sector.
- Donor agencies and intermediaries may consider developing low-cost sustainable solutions for private-sector engagement like STEPS where public program management and the private sector have equal roles and accountability.

## INTRODUCTION

India contributes to 26% of the global TB burden. With 27% of the drug-resistant TB burden, India has the largest number of drug-resistant TB patients in the world.^1^ More than half of the TB patients in India seek care from the private sector.^2^ Gaps in the TB care cascade include people with active TB not having access to correct and complete diagnosis, including diagnosis of drug resistance; people diagnosed with TB not being started on treatments; and people started on treatment not completing treatment, were observed more among patients who were diagnosed in the private sector compared to the public sector.^3^^,^^4^ There were concerns about the suboptimal quality of care including incorrect diagnosis and treatment, lack of systems for treatment adherence support, and a high loss to follow-up rate that could increase the risk of drug resistance among the patients seeking care from the private sector in India.^3^^,^^4^

The *Standards for TB Care in India* (STCI) were developed as a way to engage both the public and private sectors for effective TB prevention and control.^5^ STCI, which is a locally customized version of the *International Standards of Tuberculosis Care*, mentions 26 standards that every citizen should receive irrespective of the sector of treatment. Tools like STCI, national policy for mandatory TB notification, and NIKSHAY^6^—the case-based web-based management information system—were built to improve TB care services in the private sector in India. However, quality services as assured in the public sector, such as free diagnostics including rapid molecular tests and drug susceptibility, free drugs, treatment adherence support and monitoring, treatment location transfer, contact investigation, and TB preventive therapy, rarely reach patients who are treated in the private sector.

Conventionally, public health programs elaborate on private sector engagement and public-private partnerships. These partnership models were mostly business-centered like incentive-based and service-purchase models that were similar to a client-vendor relationship rather than an equal partnership between the public and private sectors. Many efforts have been launched to engage the private sector effectively for TB control in India.^7^^,^^8^ Several models that have successfully increased private case notifications were difficult to expand due to lesser emphasis on creating lasting partnerships and huge short-term financial implications.^9^^,^^10^

Several models that have successfully increased private case notifications are difficult to expand due to lesser emphasis on creating lasting public-private partnerships and huge short-term financial implications.

We conceived a model for ensuring standards of TB care for patients accessing the private sector called STEPS, System for TB Elimination in Private Sector. This article describes the model, its pilot implementation in the state of Kerala, and early outcomes.

## TB ELIMINATION STRATEGIES IN KERALA

With clear evidence of declining transmission of TB and lower rates of drug-resistant TB in Kerala, the Government of Kerala has declared its commitment to achieving the Sustainable Development Goals related to ending TB earlier than the rest of India.^11^ The state has notified 72 TB cases per 100,000 in 2019: 57.3 in the public sector and 14.7 in the private sector.^12^ The number of TB cases estimated to be outside the current surveillance system, estimated through TB drug sales surveillance, constituted approximately 10% of the existing TB notification.^13^

The private health care sector in Kerala accounts for more than 70% of all facilities and 60% of all inpatient beds. Most (90%) of TB notifications from the private sector used to come from the 446 private hospitals and the remaining 10% from general practitioners.

Many public-private strategies for TB control in India started in Kerala during the early years of implementation of the National TB Elimination Program (NTEP)—formerly the Revised National TB Program.^14^^,^^15^ Through a Global Fund project, since 2005, the Indian Medical Association has conducted large-scale training of private doctors in Kerala using national technical and operational guidelines and later STCI guidelines.^16^ Although this training was successful in increasing acceptance of NTEP among private practitioners, it often did not translate into large numbers of TB notification, directly observed treatment short-course (DOTS) regimen, or delivery of public health services to patients in the private sector. NTEP involvement with the private sector continued to be limited to the teaching institutions and hospitals that were managed by Indian Medical Association leaders and senior members. Even after NTEP implemented a daily anti-TB regimen—the absence of which was the major reason the private sector cited not participating—there was not much improvement in the private sector participation.^17^ Operations research identified several important barriers to engagement of the private sector in NTEP: absence of mutual trust between the public and private sector, concerns over patient confidentiality and patient choices, apprehension of losing patients, lack of consideration for hospital management, lack of time for doctors to document, inability of the program to keep commitments and timely payments, poor recognition of the private sector, and bureaucratic hurdles.^17^

A recent study from Kerala of the prescribing pattern of practitioners reported that TB management in the private sector follows a reasonable standard of care in terms of treatment prescriptions.^18^ A private tertiary care center in Kerala demonstrated that establishing a management system within the hospital ensured 100% TB notification with a 4-fold increase over 6 months.^19^ Another study that followed up a cohort of TB patients treated in private facilities in Kerala reported a loss to follow-up of 21%.^20^ Limited ability to monitor and promote treatment adherence remained a major challenge in the private sector. Although NTEP documents the treatment outcome of every patient diagnosed/enrolled for treatment, such documentation is rare in the private sector. Lack of a network of staff and providers in the private sector limits the ability to monitor and support adherence to standards of care.

There was a felt need from NTEP for innovative strategies to engage the private sector with limited resource implications and without compromising the efficiency of program management. STEPS evolved as a solution for ensuring standards of TB care in a patient-centric way for all patients accessing the private sector, addressing the concerns of the private sector. The Government of Kerala has included STEPS as 1 of the 10 key strategies in its “Kerala TB Elimination Mission” that aims to achieve Sustainable Development Goals related to TB.

## STEPS INTERVENTION MODEL FOR TB ELIMINATION IN THE PRIVATE SECTOR

STEPS is envisioned as an equal partnership between the public and private sector for the benefit of society with TB elimination as the outcome. The primary objective of STEPS is to address gaps in the quality of care for patients in the private sector by ensuring standards of TB care in both sectors to all Kerala citizens in a patient-centric manner.

**FIGURE 1:**
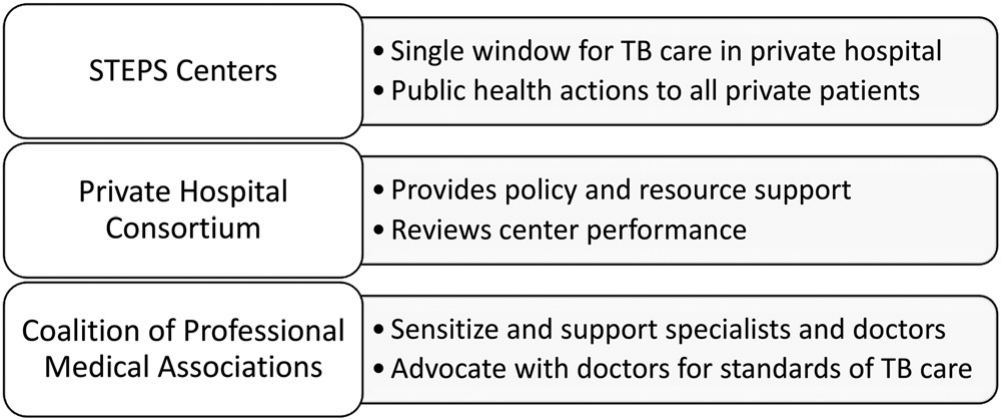
STEPS Model for Private Sector Engagement in Kerala, India Abbreviation: STEPS, System for TB Elimination in Private Sector.

The STEPS strategy has 3 components (Figure 1): (1) establish STEPS Centers, (2) form a district consortium of private hospitals, and (3) form a district coalition of professional medication associations.

### 1. Establish STEPS Centers

Establishing STEPS centers at all private hospitals was the heart of STEPS. These centers act as a single “window” in private facilities to ensure that all presumptive TB patients and the diagnosed TB patients receive standards of care. The centers provide patient notification, linkage for public health actions including contact investigations, chemoprophylaxis to eligible households, offering HIV testing, domestic airborne infection control kits, direct benefit transfers and nutritional support, and treatment adherence support.

STEPS centers act as a single “window” in private facilities to ensure that all presumptive TB patients and the diagnosed TB patients receive standards of care.

### 2. Form a Private Hospital Consortium

In every district, a consortium of private hospital owners provides policy and resource support for STEPS centers and reviews the STEPS centers' performance. Consortium members select one of the hospitals to serve as chair for a fixed term. The district program manager of NTP serves as member secretary. The consortium meets once in 3 months to review the performance of STEPS centers and suggest corrective actions if required.

### 3. Create a Coalition of Professional Medical Associations

In all districts, a coalition of professional medical associations advocates with medical practitioners and sensitizes them on STCI and STEPS. The coalition is formed under the patronage of the Indian Medical Association. Other members of the coalition included associations of chest physicians, pediatricians, general physicians, geriatrics, family medicine, nephrologists, general surgeons, orthopedic surgeons, and radiologists. Coalition members select a member from one of the associations to be coalition chair for a fixed term. The member secretary is the NTEP district program manager who provides all logistic and office support to the chair to organize the meetings, execute the decisions, and document. The coalition meets every 3 months to plan and review the activities as per the plan.

The process of establishing each STEPS component entailed the following steps:

- The NTEP district program manager, with the support of the Indian Medical Association, convened the meeting of all professional medical associations in the district to form the district coalition.
- The NTEP district program manager, with the support of the Indian Medical Association, convened the meeting of administrators of all private hospitals to form the district consortium. Every quarter, the consortium reviewed the STEPS centers' performance.
- The hospital administration established STEPS centers in the respective private hospitals and nominated individuals to serve as STEPS leads and STEPS links. The hospital management reviewed STEPS center activity in the hospital.
- The NTEP district program manager trains the STEPS leads and STEPS links.
- The NTEP district program manager and the Indian Medical Association district coordinator train members of the district coalition who, in turn, trained the member-doctors of the respective professional organizations.

**FIGURE 2:**
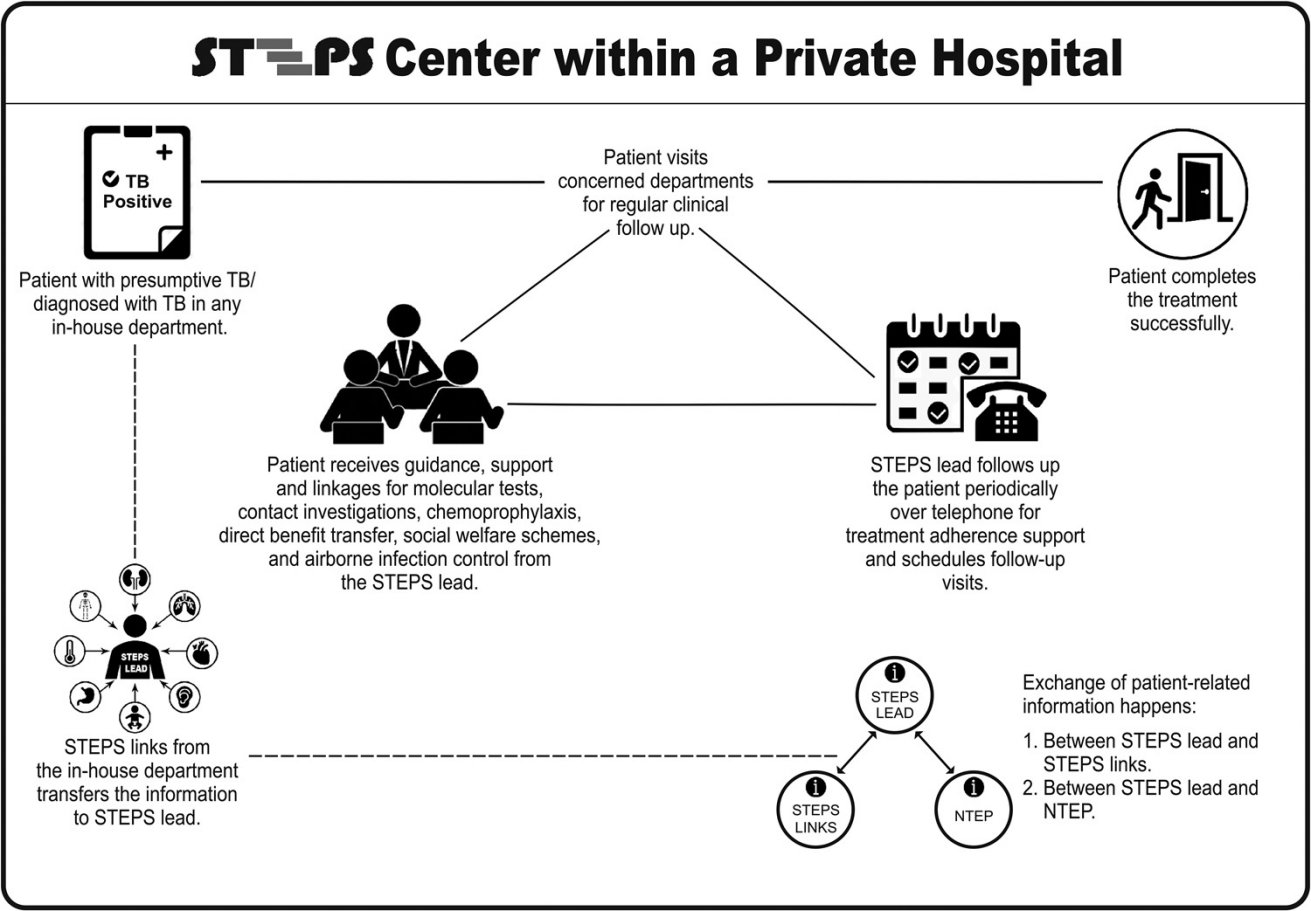
Schematic Representation of STEPS Center Within a Hospital Abbreviation: STEPS, System for TB Elimination in Private Sector.

Figure 2 shows a schematic representation of how the STEPS center functioned inside a hospital. A central person (STEPS lead), who was nominated by the hospital management, worked together with contact persons (STEPS link) for each in-house department in a hub-and-spoke model. The STEPS lead and links were typically staff nurses. The entire process fosters customer loyalty (Box 1).

BOX 1 **Fostering Customer Loyalty with STEPS**

In business, after-sales service is any support provided to a customer after the product or service has already been purchased. Companies use after-sales support as a business strategy because it typically leads to higher customer satisfaction, brand loyalty, and even word-of-mouth marketing. Similarly, a STEPS center can be viewed as an “after-sales service model” based upon a self-initiated business promotion and customer loyalty blended with the social responsibility of the private sector.

The process of STEPS centers working within hospitals fostered customer loyalty.

**FIGURE 3:**
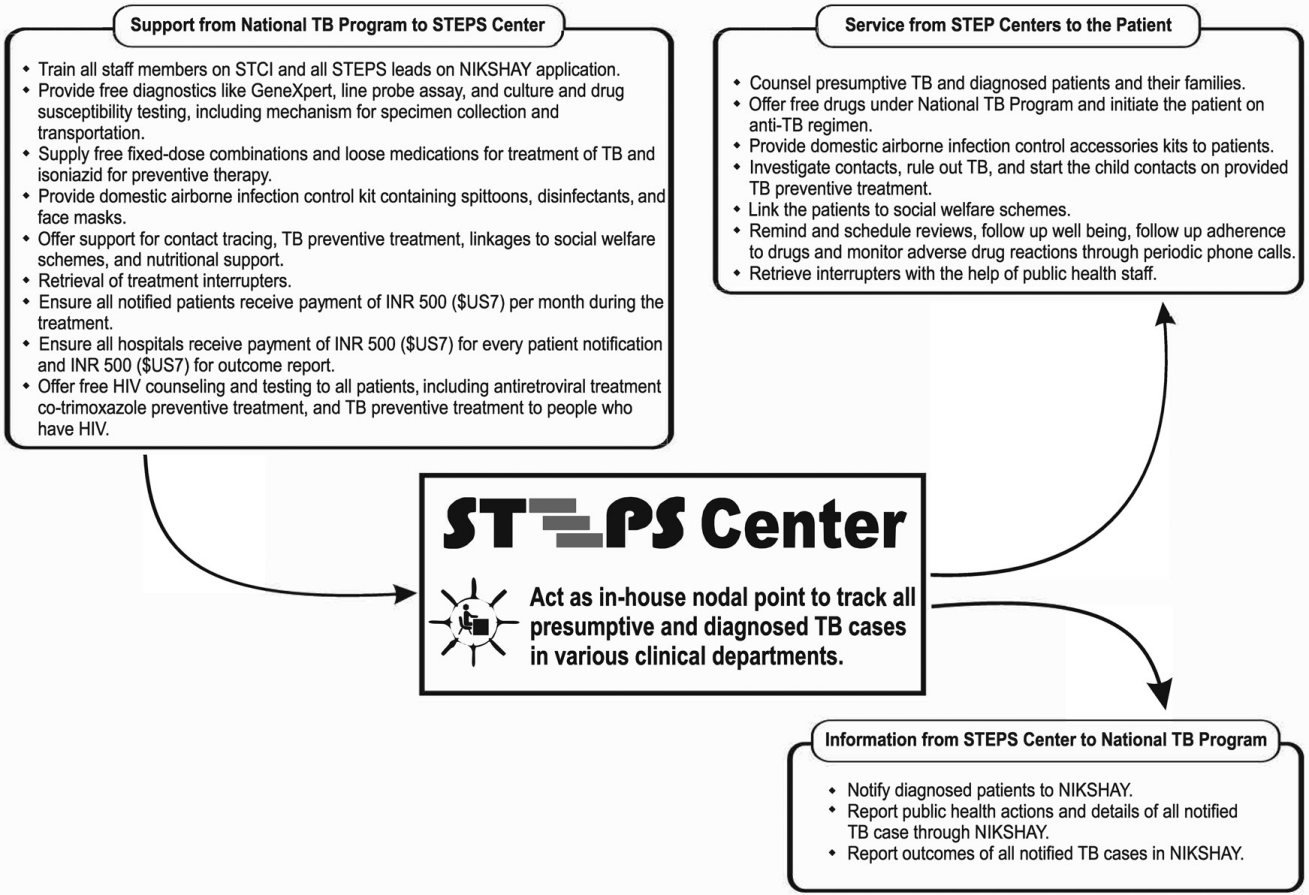
Relationship of STEPS Center with the National TB Elimination Program Abbreviation: STEPS, System for TB Elimination in Private Sector.

Based on the discussions with the hospital management, NTEP provided services customized to each hospital's demand. Figure 3 illustrates the STEPS centers' role in treating patients and tracking TB cases.

The NTEP field staff of the concerned area coordinated the NTEP services opted by the private hospitals in their area. In districts where there was a high concentration of private hospitals, additional support in terms of rearrangement of work and redeployment were done by NTEP program managers based on the workload of field staff. From the state level, the nodal officer of Public-Private Mix (PPM) coordinated the supports offered by NTEP to the various STEPS centers.

The Joint Effort for TB Elimination (JEET) project is a patient-provider support agency trying to set up effective and sustainable structures to strengthen existing systems and seamlessly extend quality TB care to patients in the private sector in India. Kerala State NTEP has customized the project and negotiated with project JEET to render the service of 1 PPM state lead and 5 city officers for 2 years to assist the NTEP district program managers in advocating with private hospital administration to start STEPS centers. They also supported the NTEP district program managers to get in touch with the professional organizations and coordinate training for STEPS leads and STEPS links. JEET staff assisted the state and district NTEP program managers in supportive supervision, reviews, capacity building, and documentation (Box 2).

BOX 2 **How Various Stakeholders Worked With the Private Sector to Establish STEPS**

National TB Elimination Program (NTEP): District program managers provided customized support to STEPS centers in private hospital based on their need through NTEP key staff. State program managers monitored, reviewed, and provided supportive supervision.

Joint Effort for TB Elimination: Rendered services of one public-private mix lead and 5 city officers for 2 years to assist NTEP program managers in establishing STEPS and act as interphase between NTEP and private sector.

Indian Medical Association: Coordinated specimen collection and transportation system from major private hospitals through a partnership scheme with NTEP, facilitated formation of coalition of professional organization in every district, and served as ambassadors for STEPS.

World Health Organization NTEP Technical Support Network: Developed the idea of STEPS, coordinated the process, and technically supported all stakeholders in establishing STEPS.

Through a memorandum of understanding, NTEP has partnered with Indian Medical Association and established a specimen collection and transportation system from the major private hospitals in the state to public GeneXpert sites.

## EARLY RESULTS

STEPS was initiated in January 2019. Each of the 14 districts formed a private hospital consortium and a coalition of professional medical associations. Each coalition and consortium conducted a minimum of 2 meetings during 2019.

Of the 446 hospitals mapped, 318 established STEPS centers during 2019. The hospitals that reported the most TB patients were targeted at the beginning of the pilot for onboarding to STEPS. The remaining 128 hospitals established STEPS in 2020.

Since the STEPS model was piloted, TB patient notification to NTEP from the private sector in Kerala has increased by 26% in 2019 compared to 2018 from 3,981 cases to 5,003; 78% of these notifications were contributed by the STEPS centers.

Since the STEPS model was piloted, TB patient notification to NTEP from the private sector in Kerala increased by 26% in 2019.

The Table compares TB program indicators before (2018) and after (2019) implementing the STEPS model.

**TABLE:** Comparison of National TB Program Indicators Before and After Implementation of STEPS Model in Kerala, India

Indicator	2018	2019	Percentage Change
TB cases notified by the private sector, no.	3,981	5,003	+26%
Private specimens tested by public GeneXpert machines, no.	7,872	14,210	+ 81%
Microbiologically confirmed cases among notified TB, no. (%)	995 (25%)	1,951 (39%)	+ 56%
Patients notified of TB test results, no. (%)			
Patients tested for rifampicin resistance at baseline with GeneXpert	637 (16%)	1,951 (39%)	+143%
TB patients who know their HIV status	1,672 (42%)	4,102 (82%)	+95%
Patients received Direct Benefit Transfer	1,273 (32%)	3,552 (71%)	+122%
Patients whose treatment outcome was reported in Nikshay	1,393 (39%)	4,312 (86%)	+120%
Patients with TB treatment outcome reported, no. (%)			
Patients' treatment was completed successfully	1,253 (90%)	3,797 (88%)	−2%
Patients lost to follow-up	31 (2%)	87 (2%)	0%

STEPS led to a shift from using private anti-TB drugs to NTEP-supplied drugs, leading to 2,000 additional cases being put on NTEP-supplied drugs. Overall, 70% of all cases notified from the private sector in 2019 were treated with NTEP-supplied drugs. Data officially collected by the state drug controller showed that sale of anti-TB drugs decreased from 1.6 million rifampicin units in 2018 to 0.5 million rifampicin units in 2019 after the establishment of the STEPS model.

## KEY CHALLENGES FACED DURING IMPLEMENTATION

### Changing Attitudes

The biggest challenge faced was changing the attitude of the public program managers and staff. Public program managers conventionally preferred to wear a “policing” cap and an “authoritarian” attitude as far as private sector engagement was concerned. Engaging the private sector has never been fully owned by the public sector. Private hospitals that do not follow NTEP were considered to be “enemies” of the program. Change management strategies were devised for changing these attitudes among public program managers. Rather than using technical concepts to change these attitudes, we promoted the idea that engaging the private sector will help the patient, who is ultimately the Government's responsibility.

Convincing private hospital management to participate in the STEPS model was challenging. To change the private sector providers' attitudes that their engagement meant pulling them toward the public program, we instead emphasized that the private sector would be enabled to ensure standards of care through sensitization, training, communication, and supportive supervision visits.

### Advocating for the STEPS Strategy

When the STEPS strategy was clearly communicated to hospital management, they were willing to start STEPS centers at their hospitals. Encouraging social responsibility to fight a disease in a business model by fostering customer loyalty without much interference by external agencies was more than enough reason for the hospital management to join the “movement.” The leaders who started STEPS centers in their hospitals were made advocates for convincing others. The STEPS model was socially marketed among the private sector as a model owned by private hospitals to help the hospitals to ensure standards of care to their patients. Only the concepts were marketed, and the hospitals were given the flexibility to customize the model within their hospitals to meet the objectives. During the initial year, 2–3 visits by city officers of Joint Effort for TB Elimination and NTEP program staff were required to convince each hospital management to start STEPS centers. However, later many private hospital managements started contacting the NTEP program managers to enroll in STEPS

### Personnel-Related Issues

Gaining the trust of some doctors in the private sector was a challenge in the beginning. Local leaders of the Indian Medical Association and the coalition of professional medical associations have played the role of ambassadors. When the doctors were convinced that STEPS was a system to ensure the standard of care to their patients, which addressed all their prior concerns with NTEP, most of the providers accepted the program.

Public sector staff at the district level felt apprehensive and lacked the confidence to talk to corporate hospital management staff. The professional approach used by the 5 trained city officers of the Joint Effort for TB Elimination project helped in liaising between the public and private partners.

Personal conflicts between staff of both sectors were observed in very few instances. Early identification of the problems due to open, proactive, and vigilant systems in the program at the district and state level helped troubleshoot those issues promptly.

Conscious efforts were made to sustain the motivation of private sector staff nurses, doctors, and management. WhatsApp groups with all STEPS leads and program staff in every district not only facilitated easy communication and cross learnings but also offered an opportunity to share their success. A letter of appreciation from the highest government officials was sent to all private hospitals' management. Private hospital representatives were involved right from the planning phase to implementation and evaluation. Because the model promoted local customization by private hospitals, the key hospital staff were encouraged to keep working on improving the model within their hospital, which fostered a feeling of ownership.

NIKSHAY, the web-based case surveillance system of NTEP was used to try to document the outcomes and endpoints of various services received by every patient. A uniform web-based system for documenting the process of how a patient received those services has yet to be established.

## DISCUSSION

The National Strategic Plan (NSP) for TB Elimination 2017–2025 emphasizes the government's role as an enabler and not as the sole provider of TB care. NSP envisions ensuring that services are established as per *Standards for TB Care in India* to privately managed patients.^21^

The STEPS strategy, which is aligned with the NSP, helped to improve standards of care for patients reaching the private sector, reduce out-of-pocket expenditure for tests and drugs, and strengthen the health system to ensure universal access to TB care. STEPS extended the full range of program services to the patient in a verifiable way. STEPS demonstrated that low-cost locally customized private sector engagement models with good administrative commitment are feasible and beneficial to society. STEPS also demonstrated how the Joint Effort for TB Elimination project, a private provider support agency, added value in developing sustainable systems for private sector engagement by customizing a collaborative and monitoring framework.

STEPS demonstrated that low-cost locally customized private sector engagement models with good administrative commitment are feasible and beneficial to society.

After implementing the STEPS model, improvements in documented quality of care were evident. The model ensured that almost all TB patients who sought care in the private sector had a documented treatment outcome and demonstrated improvements in obtaining microbiological confirmation of TB to correctly diagnose TB and in offering testing for drug-resistant TB at baseline itself. This effort is extremely important for India where the proportion of drug-resistant TB is higher than in other countries. Similarly, the proportion of TB patients in the private sector who knew their HIV status and those who received financial benefits also improved.

STEPS fostered customer loyalty between the patient and the private hospital without any interference from external agencies. Loyal patients will in turn bring back business to the hospital. Return business might have motivated some of the corporate hospitals to appoint a full-time staff person to work with the STEPS centers. The financial incentives provided by NTEP to the hospital ($US7 per notification and $US7 per treatment outcome) were used for establishing the systems within the hospital, including some appointing a new staff person. A few hospitals started using the STEPS model for patients receiving treatment for diseases other than TB. In the future, the strategy may inculcate a culture among private hospitals of ensuring public health actions to their patients. STEPS may also lead the way for establishing a system for total engagement of the private sector for all national health programs other than TB.

The STEPS model has no additional financial or human resource implications to the program. It simply tried to optimize the already existing resources and commitments from NTEP. It relied on the social responsibility of the private sector combined with providing profitable customer care services and realizing the Government's actual role in enabling TB services to the entire population, including those who seek care in the private sector. It is a win-win-win-win situation for the patient, NTEP, private hospitals, and the society, rendering itself easily sustainable and replicable. The model could be locally customized to any setting.

While implementing STEPS, we learned many new lessons. Private hospitals have been very willing to be part of public health initiatives. Developing a public health outlook for private hospitals is possible. This may apply not only to TB but also to many health programs including other communicable diseases and maternal and child health. Involving private hospital management during the whole process was the key to success. When the upper-level management staff are convinced, most of the local problems had easy solutions. To cite an example, many private hospital management groups decided to let their in-house pharmacies provide (private) anti-TB drugs to patients only after notification to NIKSHAY, ensuring 100% real-time notification. Allowing local customizations of the model fosters ownership, which itself sustains their motivation. We left STEPS as an “open system,” so that many improvements emerged. One such example is the model of a digitalized STEPS register, which was developed by one of the staff nurses from a private hospital.

The key reason that STEPS was successful was the clear understanding and delineation of the roles, responsibilities, and accountabilities of both public and private sectors based on each stakeholder's skills and expertise. The partnerships were customized for each private hospital. Having strong political and administrative commitment from the state program managers and training succeeded in changing the attitude of the NTEP staff toward the private sector. An important strategic change that contributed to rapid scale-up and acceptance of STEPS was the attempts to gain the trust of hospital management, involvement of nurses for counseling, efforts for quality control through a coalition of professional medical associations, lack of formal memoranda of understanding, and lack of major financial transactions between partners.

Gaining trust of hospital management, involving nurses for counseling, and establishing quality control through professional medical organizations helped STEPS be rapidly scaled up and accepted.

A joint monitoring mission held in India in November 2019 led by the World Health Organization and global developmental partners, including 165 multidisciplinary professionals from technical agencies, national institutes, medical colleges, and civil society, recommended supporting the establishment of STEPS Centers in all private health care facilities along with a formal evaluation to inform its expansion to other states.^22^ A formal evaluation including economic evaluation is being conducted now.

The current model has an anticipated minor risk of interruption or delay in supply of NTEP products to private hospitals due to the workload of NTEP staff or due to personal conflicts. Separate systems for supply chain management to private hospitals, online pharmacies, and vouchers for drugs that flow through the private supply chain could be some of the solutions that could mitigate this risk. Also, the public sector may not be able to address the entire diagnostic access demands for patients reaching the private sector in the long run. Schemes like public purchasing of private laboratory services may need to be established. The current experiences with the STEPS model are only with private hospitals and not with individual practitioners.

In summary, STEPS is a low-cost private sector engagement model which helped to ensure standards of care to patients reaching the private sector. The model, which relies on the social responsibility of the private sector and self-realization of the public sector about its actual role to engage the private sector in serving their citizens, was beneficial to society. Locally customized STEPS could be one of the major solutions for supporting TB patients reaching the private sector.

## Notes

**Peer Reviewed**

**First published online:** May 27, 2021.**Cite this article as:** Balakrishnan S, Rakesh PS, Sunilkumar M, et al. STEPS: a solution for ensuring standards of TB care for patients reaching private hospitals in India. *Glob Health Sci Pract*. 2021;9(2):286-295. https://doi.org/10.9745/GHSP-D-20-00449
